# Education and employment status of adults with autism spectrum disorders in Germany – a cross-sectional-survey

**DOI:** 10.1186/s12888-018-1645-7

**Published:** 2018-03-27

**Authors:** Fabian Frank, Martina Jablotschkin, Tobias Arthen, Andreas Riedel, Thomas Fangmeier, Lars P. Hölzel, Ludger Tebartz van Elst

**Affiliations:** 1 Department of Psychiatry and Psychotherapy, Medical Center – University of Freiburg, Faculty of Medicine, University of Freiburg, Hauptstraße 5, 79104 Freiburg, Germany; 2 Department of Social Work, Protestant University of Applied Sciences Freiburg, Bugginger Straße 38, 79114 Freiburg, Germany; 3 Department of Oncological Rehabilitation – UKF Reha gGmbH, Medical Center – University of Freiburg, Breisacher Straße 117, 79106 Freiburg, Germany; 4 Parkklinik Wiesbaden Schlangenbad, Rheingauer Straße 47, 65388 Schlangenbad, Germany

**Keywords:** Autism, Asperger syndrome, High functioning, Employment, Education, Adults

## Abstract

**Background:**

Adults with autism spectrum disorders (ASD) experience challenges in participating in the labour market and struggle to achieve and maintain appropriate professional positions, possibly due to impairments of communication and social interaction. Studies have shown high rates of unemployment as well as evidence of inadequate employment. As knowledge on the participation in the German labour market is scarce, the aim of our study was to examine employment status, type of occupation and inadequate employment in a sample of clinically mostly late-diagnosed and most likely not intellectually disabled adults with ASD in Germany.

**Methods:**

We conducted a cross-sectional-survey in clinically mostly late-diagnosed adults with ASD. Employment status, type of occupation, and the level of formal education and training were examined through a postal questionnaire. Inadequate employment regarding participants’ current and longest practised occupation was assessed by transforming participants’ information into skill levels of the “Classification of Occupations 2010” of the German Federal Employment Agency, and comparing these with participants’ level of formal education and training.

**Results:**

The response rate was 43.2% (*N* = 185 of *N* = 428 potential participants). 94.6% were first-time diagnosed when being 18 years of age or older. 56.8% held a general university entrance-level qualification and 24.9% had obtained a Masters’ or diploma degree as their highest vocational qualification. 94.1% had been employed at some time. Of these, 68.4% reported being currently employed, 13.5% being currently unemployed and 17.0% being retired for health reasons. Regarding the longest-practised and the current occupation, the highest proportion of participants was found in the occupational area “health and social sector, teaching and education” (22.4% and 23.3%, respectively). With respect to inadequate employment, 22.1% were found to be overeducated in relation to their longest-practised occupation and 31.3% in relation to their current occupation. This is significantly higher than the percentage of overeducation in the general population.

**Conclusions:**

Despite largely high formal qualifications, the clinically mostly late-diagnosed adults with ASD represented in our sample are disadvantaged regarding their participation in the German labour market, especially with respect to rates of unemployment, early retirement and overeducation. Employment support programs should be developed to improve employment outcomes.

**Electronic supplementary material:**

The online version of this article (10.1186/s12888-018-1645-7) contains supplementary material, which is available to authorized users.

## Background

Autism spectrum disorders (ASD) are associated with qualitative impairment in using and contextualizing communication for social purposes and the capacity to process socially relevant information, and with restrictive repetitive behaviour patterns [1, 2]. Given that the capacity to process socially relevant information and to interact in a socially suitable manner are essential requirements in occupational environments, everyday work processes can pose major challenges for adults with ASD [3], as most workplaces require adherence to social norms and decorum. With respect to social and sensory issues interfering with their job performance [4] as well as potential problems regarding their ability to manage social and interactional aspects of work [5, 6], earlier studies revealed challenges in obtaining, securing and maintaining employment in adults with ASD [7–9]. Furthermore, successful participation in the labour market can be hindered by differences with employers, for example regarding the understanding of productivity requirements or required support in work [10], and by possible ASD-related problems, for example prioritization and self-organization of work-tasks.

Even in adults with ASD and no co-occuring intellectual disability, studies show high rates of unemployment up to 60% [4, 5, 8, 9, 11–14]. In this context, it is important to recognize that adults with ASD are often high educational achievers in terms of school and university qualifications [12, 13] and therefore apparently have good prerequisites for adequate participation in the labour market. However, a recent Australian study by Baldwin et al. [15] showed that 46.2% of employed adults with ASD were inadequately employed or overeducated, meaning that their highest level of formal education and training exceeded the occupational skill level needed for their current occupation. These findings indicate that even though individuals with ASD often possess high levels of formal education and training [12, 15] and desire to work [16], adults with ASD often struggle to participate in the labour market or to achieve and maintain appropriate professional positions.

Apart from studies reporting rates of 60% not-working (employable age, but no occupation due to i.e. being retired for health reasons, being homemaker or being unemployed) [12] or of 36.0% being unemployed [4] knowledge on the participation of adults with ASD in the German labour market in terms of adequate employment is scarce. Therefore, the aim of our study was to examine, by means of a cross-sectional-survey, the integration of a sample of clinically mostly late-diagnosed and most likely not intellectually disabled adults with ASD in the German labour market in terms of employment status, type of occupation and, in particular, regarding adequate or inadequate employment in terms of over- or undereducation.

## Methods

### Design and sample

The study population of the present postal cross-sectional-survey consists of a sample of clinically mostly late-diagnosed and most likely not intellectually disabled individuals with ASD who attended the “specialised outpatient assessment clinic for ASD in adulthood” (Spezialsprechstunde für Autismus-Spektrum-Störungen im Erwachsenenalter) of the Medical Center – University of Freiburg for diagnostic analysis between September 2009 and March 2014. Clinically late-diagnosed means, that ASD was first time diagnosed when individuals were in adult age of 18 years or older. Diagnostic analysis was based on a structured clinical examination following ICD-10 and DSM-IV criteria, the NICE-Guidelines [17] and the German S3-Guidelines for diagnosing ASD [18]. A detailed description of the diagnostic approach and of the procedures and measures used is provided elsewhere [12, 19]. All individuals aged 18 years or older diagnosed with ASD during this consultation between September 2009 and March 2014, who did not object to being contacted for research purposes, were included in the study. Formal IQ-testing was not part of the diagnostic process in all of these individuals. Of those individuals who have undergone formal IQ-testing, no individual possessed an IQ-score below 70. Furthermore, based on the educational performance and other assessments, we do not except any of the diagnosed individuals to be ranged as intellectually disabled. Therefore, it is most likely, that there are no individuals with co-occuring intellectual disability represented in our initial sample. Between September 2009 and March 2014, *N* = 485 individuals were diagnosed with ASD. *N* = 57 objected being contacted for research purposes. Therefore, the potential study population comprised *N* = 428 adults with ASD. In October 2015 these individuals received information on the study and a questionnaire by post. A post-paid and post-addressed return envelope was enclosed, and a reminder letter was sent to all potential participants after two weeks, as these measures are associated with an increased response rate [20]. The survey was conducted with full anonymity and participation was voluntary. The study was approved by the Ethics Review Committee of the Medical Centre – University of Freiburg (number: 288/15).

### Measures and procedures

Regarding the level of formal education and training, the employment status, the type of occupation, and the occupational skill level, the following measures were obtained or derived from participants.

#### Employment status

Initially, participants were asked whether they had ever been employed (yes/no). Thereupon, participants who had ever been employed were asked whether they were employed at present (yes/no) and, if so, how many hours they usually worked per week and whether their employment was located in a sheltered environment (yes/no). Additionally, participants who had ever been employed were asked whether they rated themselves as currently unemployed (yes/no), for how many months they had been unemployed within the last five years, and whether they had already retired (yes/no), and for which reasons (age, early retirement, health reasons).

#### Type of occupation

To classify the occupation type for the current and the longest practised occupation, the survey asked participants to record (a) their professional activity, (b) their subject area, and (c) their economic sector within short open-ended questions for both, the current and the longest practised occupation. This information was used to assign each participant to an occupational grouping according to the “Classification of Occupations 2010” (Klassifikation der Berufe 2010; KldB) of the German Federal Employment Agency [21]. The KldB codes all jobs and occupations in the German labour market by means of a five-digit documentation code. The first digit describes the occupational area, the second indicates the major occupational group, the third represents the sub-major occupational group, the fourth shows the minor occupational group, and the fifth describes the occupational skill level [21]. To ensure a consistent transformation of participants’ occupational information into the KldB classification, an online database of the German Federal Employment Agency was used [22]. The occupational information recorded by the participants was entered into this database and automatically transformed into the five-digit documentation code of the KldB. Additionally the derived KldB five-digit documentation codes of every participant were manually reviewed for plausibility and adjusted if necessary. An explanatory example for the transformation of participants’ information into KldB five-digit documentation codes – in this case the documentation code for a medical specialist in psychiatry – is given in Table 1.

**Table 1:** Example for transformation of participants’ information into KldB codes

Description of occupation within the survey					
survey Item	professional activity	subject area		economic sector	
example	physician	psychiatry		health care system	
Transformation in 5-digit documentation code of the KldB [21, 22]					
structure	occupational area	occupational group			occupational skill level
		major	sub-major	minor	
5-digit code	8	1	4	6	4
explanation	health and social sector	medical health	human and dental medicine	psychiatric consultant	highly complex occupational activities

To assess differences from the general population, participants’ data regarding the occupational area (first digit of the KldB five-digit documentation codes) of the current occupation was compared to data from the German Federal Employment Agency [23].

#### Occupational skill level and level of formal education

The fifth digit of each occupation listed within the KldB describes the occupational skill level. The KldB comprises four skill levels, which are differentiated as follows: level 1 – assistant or semi-skilled occupational activities; level 2 – professional oriented occupational activities; level 3 – complex specialist occupational activities; level 4 – highly complex occupational activities [23]. According to the Institute for Employment Research (Institut für Arbeitsmarkt- und Berufsforschung) of the German Federal Employment Agency skill levels are primarily defined by the required level of formal education and training [24], as shown in Table 2.

**Table 2:** Formal level of education and training transferred to KldB

Formal level of education	KldB occupational skill level	
Master’s degree or Diploma university	4	highly complex occupational activities
Master’s degree or Diploma university of applied sciences		
Bachelor’s degree: university or university of applied sciences/technical college	3	complex specialist occupational activities
Apprenticeship II: technical school, master school or vocational academy		
Apprenticeship I: dual system of vocational and educational training within vocational schools and/or companies	2	professional orientated occupational activities
Other vocational qualification	1	assistant or semi-skilled occupational activities
No vocational qualification		

For each participant, the occupational skill level was recorded based on the previously derived KldB five-digit documentation code. To assess inadequate employment, i.e. over- or undereducation in relation to the current and the longest-practised occupation, we compared the skill level of the related KldB documentation code with the skill level derived from the level of formal education and training. Inadequate employment in terms of overeducation is assumed if a lower KldB skill level is accompanied by a higher KldB level of formal education and training. Conversely, undereducation is indicated if a higher KldB skill level is accompanied by a lower KldB level of formal education and training. Overeducation and undereducation were examined for both the longest-practiced occupation and the occupation pursued at the time of the survey. To assess differences compared to the general population, participants’ data regarding over- or undereducation with respect to the current occupation was compared to data derived from the employment statistics of the German Federal Employment Agency [25].

#### Sociodemographic and clinical variables

Sociodemographic data (age, sex, marital and relationship status, school-leaving qualification) and presence of comorbidities were collected using the German Health Interview and Examination Survey for Adults [26]. Additional clinical variables (ASD diagnosis, age at first-time ASD diagnosis) were collected using self-constructed items.

#### Pretest

Since individuals with ASD often have peculiarities in their understanding and use of language [27], impairment of linguistic pragmatics was considered in the construction of the questionnaire. Therefore, the endeavor was to use comprehensible and unequivocal formulation, which was examined within a pretest in five adults diagnosed with ASD using think-aloud methodology [28]. The questionnaire was then adapted linguistically and visually based on the results of this pretest. The final version of the questionnaire is available in German as a supplementary file (see Additional file 1).

#### Statistical analysis

Descriptive data analysis was performed using IBM SPSS Statistics for Windows, version 21. Comparisons of participants’ data with data from the general population were conducted using Chi-square tests.

## Results

The response rate was 43.2% (*N* = 185 of *N* = 428 potential participants). Participants’ sociodemographic and clinical characteristics are summarized in Table 3.

**Table 3:** Sociodemographic and clinical characteristics

Sociodemographic Characteristics			
Gender (*N* = 185)		Family status (*N* = 183)^d^	
male^a^	61.6	unmarried^a^	71.0
Age (*N* = 185)		married^a^	22.4
mean (SD)^b^	39.5 (11.3)	divorced^a^	6.6
minimum^c^	21	Relationship status (*N* = 183)^d^	
maximum^c^	64	long-term relationship > 6 months^a^	36.6
Clinical Characteristics			
Primary Diagnosis (*N* = 185)		Comorbidities^a,e,f^ (*N* = 184)	70.1
asperger^a^	78.9	Depression^a^	48.9
atypical^a^	9.7	orthopedic diseases^a^	19.0
infantile^a^	4.3	OCD^a^	13.0
not specified / unknown^a^	7.1	respiratory diseases^a^	11.4
Age when diagnosed I (*N* = 184)^e^		Age when diagnosed II (*N* = 184)^e,g^	
mean (SD)^b^	34.7 (12.5)	≥ 18 years of age^a^	94.6
minimum^c^	3	≤ 18 years of age^a^	5.4
maximum^c^	60		

^a^in percent

^b^means and standard deviation

^c^years of age

^d^missing data *N* = 2

^e^missing data N = 1

^f^just the four most stated comorbidities are listed

^g^being ≥18 years of age at first-time diagnosis is classified as late-diagnosed

The majority of the participants is male (61.6%) and the mean age of the sample was 39.5 (SD 11.3) years (c.f. Table 3). 36.6% were in a long-term relationship. The most frequent self-reported ASD diagnosis was asperger’s syndrome (78.9%), while the mean age at first-time diagnosis of any ASD was 34.7 (SD 12.5) years. 94.6% were 18 years of age or older at first-time diagnosis of any ASD and hereby can be classified as clinically late-diagnosed. The most frequent self-reported comorbidity was depression, at 48.9% (c.f. Table 3).

### Level of formal education and training

Regarding the general education level, 11.9% of the participants held a certificate of basic secondary education (German: Hauptschulabschluss; 9 years school attendance; lower secondary level), 28.6% had a general certificate of secondary education (German: Realschulabschluss; 10 years school attendance; lower secondary level), 56.8% had a general university entrance-level qualification (German: Hochschulreife; 12 or 13 years school attendance; upper secondary level), and 2.7% had no school-leaving certificate or had a qualification that was not further specified (missing data *N* = 0).

With regard to the level of formal education and training according to the KldB, 13.3% of the participants were assigned to skill level 1 (10.5% no vocational qualification; 2.8% other vocational qualification), 43.1% to skill level 2 (43.1% apprenticeship I), 12.7% to skill level 3 (8.8% apprenticeship II; 3.9% Bachelor’s degree), 24.9% to skill level 4 (8.8% Master’s or diploma degree from university of applied sciences; 16.0% Master’s or diploma degree from university), and 6.1% were still in vocational education and training, meaning that their skill level was not specified within the KldB classification (missing data *N* = 4).

### Employment status

Regarding the employment status, 94.1% (*N* = 174) of the participants reported having ever been employed (missing data *N* = 0). Of these, 68.4% (*N* = 119) reported being employed at present, with an average number of 33.3 h worked per week (SD 12.1; missing data *N* = 6) and 8.0% reported working in a sheltered workshop (missing data N = 6). Regarding the current employment situation of participants who had ever been employed, 17.0% were in early retirement for health reasons (missing data *N* = 3) and 13.5% stated that they were unemployed (missing data N = 3). Of those who had ever been employed, 49.0% reported no periods of unemployment in the five years prior to this survey (missing data N = 17). Participants with periods of unemployment within this time frame (*N* = 80) reported an average of 24.4 (SD 20.0; Range 0.50 to 60.00) months of unemployment in the last five years (missing data N = 0).

### Type of occupation

Apart from the occupational area “armed forces”, all KldB occupational areas were represented in the study sample (c.f. Table 4).

**Table 4:** Occupational area by KldB

Occupational area by KldB	longest- practiced occupation (*N* = 161)^b^	current occupation (*n* = 103)^c^	occupation general population [23]^d^
Agricultural, forestry and horticulture sector^a^	1.2	1.0	1.5
Commodity extraction, production and manufacturing^a^	16.1	12.6	22.5
Construction, architecture, surveying^a^	2.5	3.9	5.8
Natural sciences, geography and informatics^a^	13.7	15.5	3.6
Traffic, logistics, protection and security^a^	9.3	9.7	13.2
Commercial services, commodity trading, sale, tourism^a^	9.3	8.7	12.1
Corporate organization, accounting, legal and administrative sectors^a^	19.3	19.4	20.6
Health and social sector, teaching and education^a^	22.4	23.3	17.7
Linguistics, literature, humanities, social and economic sciences, media, art, culture and design^a^	6.2	5.8	2.7

^a^in percent

^b^missing data *N* = 6; not classifiable *N* = 7

^c^missing data *N* = 7; not classifiable *N* = 9

^d^no data available for the occupational area “armed forces”

Regarding the longest-practised and the current occupation, the highest proportion of participants was found in the occupational area “health and social sector, teaching and education” (22.4% and 23.3%, respectively) followed by “corporate organization, accounting, legal and administrative sectors” (19.3% and 19.4%, respectively). A comparison of participants’ distribution across the various occupational areas (regarding their current occupation) with data from the German Federal Employment Agency [23] revealed a significant difference between the study sample and the general population (Chi^2^ = 54.0; df 8; *p* < .001). In particular, the proportion of participants working in the field of “natural sciences, geography and computer science” was higher than in the general population (c.f. Table 4).

### Occupational skill level, inadequate employment and overeducation

To assess inadequate employment in terms of over- or undereducation in participants who had ever been employed (*N* = 174) not including education and training (*N* = 9), a vocational orientation phase (*N* = 1), or a so-called one-euro-job (*N* = 1; one-euro jobs are public services jobs that pay at least one euro per hour and do not affect unemployment benefits in individuals who are registered as unemployed), the KldB skill levels with respect to the longest-practised occupation were compared with participants’ highest level of formal education and training according to the KldB skill levels (missing data *N* = 9). Likewise, for participants who were employed at the time of the survey (*N* = 119), not including education and training (*N* = 8), retraining (*N* = 1) or an internship (*N* = 1), the KldB skill levels with respect to the current occupation were also compared with participants’ highest level of formal education and training according to the KldB skill levels (missing data *N* = 10).

In Table 5, the column “all” shows the distribution of participants across KldB skill levels regarding their longest-practised and their current occupation. For both, the majority of participants were situated in skill level 2 (50.6% and 41.4%, respectively). The following columns in Table 5 sort participants into three categories relating to the match or mismatch between the skill level of their longest-practised respectively current occupation and their level of formal education and training according to the KldB. In total, 62.3% were found to be at parity regarding the skill requirements of their longest-practised occupation, 15.6% were undereducated, meaning that their skill levels of formal qualification were below the occupational skill requirements, and 22.1% were classified as overeducated (c.f. Table 5). With respect to the current occupation 54.5% were found to be in parity, 14.1% were undereducated and 31.3% were classified as overeducated, meaning that their formal qualifications exceeded the skill requirements needed for the current occupation (c.f. Table 5).

**Table 5:** Occupational skill level, overeducation and undereducation

skill level longest-practiced job^a^		all	parity	undereducated	overeducated
1	n	16	4	–	12
	%	10.4	25.0	–	75.0
2	n	78	56	7	15
	%	50.6	71.8	9.0	19.2
3	n	21	6	8	7
	%	13.6	28.6	38.1	33.3
4	n	39	30	9	–
	%	25.3	76.9	23.1	–
column total	n	154	96	24	34
	%	100	62.3	15.6	22.1
skill level current job^b^		all	parity	undereducated	overeducated
1	n	17	3	–	14
	%	17.2	17.6	–	82.4
2	n	41	26	2	13
	%	41.4	63.4	4.9	31.7
3	n	11	1	6	4
	%	11.1	9.1	54.5	36.4
4	n	30	24	6	–
	%	30.3	80.0	20.0	–
column total	n	99	54	14	31
	%	100	54.5	14.1	31.3

^a^*N* = 154; missing data *N* = 9; excluded because of being in education and training *N* = 9, excluded because of stating the longest practiced occupation being a vocational orientation phase *N* = 1 or a so-called one-euro-job *N* = 1

^b^*N* = 99; missing data *N* = 10; excluded because of being in education and training *N* = 8, excluded because of stating the present practiced occupation being retraining *N* = 1 or an internship *N* = 1

According to data from the employment statistics of the German Federal Employment Agency, 63.0% of the general population in Germany are at parity, 22.0% are undereducated and 15.0% are overeducated in their employment [25]. In this regard, there is a significant difference between the general population and the study sample with respect to under- and overeducation regarding the current occupation (Chi^2^ = 21.0; df 2; *p* < .001).

## Discussion

This study aimed to examine the participation of clinically mostly late-diagnosed and most likely not intellectually disabled adults with ASD in the German labour market by means of a postal cross-sectional survey in former patients of the “specialised outpatient assessment clinic for ASD in adulthood” of the Medical Center – University of Freiburg.

A sufficient response rate of 43.2% was achieved. However, as not all potential participants replied, the critical issue is the representativeness of this sample. It remains unclear whether individuals with, for instance, higher psychosocial functioning or better opportunities and achievements in the labour market were more likely to participate in this survey, which might have biased the results. It is most probable that the employment rate and appropriate occupational positions were overestimated, as it is well known in research that individuals are more likely to report positive aspects than negative aspects [29].

Although the majority of our sample was male, the proportion of women was higher than in the typical male-to-female ratio in ASD [30]. However, this ratio is in accordance with comparable studies, which showed higher proportions of women among late-diagnosed adults with ASD [12, 13, 31]. This might be explained by gender specific compensation strategies [32], for example imitation learning, which lead for example to a better social adjustment and therefore a later identification of ASD. Thus, the late first-time diagnosis in 94.6% of the participants in this sample can be deemed as a specificity of this study. Our sample probably represents a subsample of adults with ASD comprising more able individuals with comparatively good adaptive and compensatory social skills. This is also reflected in the specificity of our study design and sample, addressing adults with clinically mostly late-diagnosed ASD and most likely no co-occuring intellectual disability. As a consequence, this might also explain the high proportion of participants who were married or in a long-term relationship compared to other studies on psychosocial outcomes in adults with ASD [13, 33, 34]. However, high rates of self-reported comorbidities were found, comparable to those reported in other studies [13, 33], which might also influence participants’ employment situation. All participants were at an employable age, with an age range of 21 to 64 years, and therefore represent the relevant target group of this survey.

Regarding the general level of education, the sample considerably exceeds the educational attainment of the general population, with 56.8% of participants possessing university entrance-level qualifications, compared to 29.5% within the general population in Germany in 2015 [35]. In Germany the university graduate ratio was 32.0% in 2014 [36], and 14.8% of the general population possessed a Masters’ or diploma degree as their highest vocational qualification in 2015 [35] and would therefore be assigned to skill level 4 of the KldB in terms of the level of formal education and training. This falls well below the percentage of study participants in our sample assigned to skill level 4 (24.9%). As the proportion of participants with no qualifications (10.5%) or a not further specified qualification (2.8%) who can therefore be assigned to KldB skill level 1, is also below that of individuals with no vocational qualifications in the general population in Germany (16.8%) in 2015 [35], our sample shows a comparatively high level of formal education and training in adults with late-diagnosed ASD, which potentially leads to promising chances in the labour market. In this respect – at first glance – participants’ participation in the labour market seems satisfactory, as only 5.9% had never been employed. However, only 68.4% of those who had ever been employed were currently employed at the time of the survey. Moreover, a self-reported unemployment rate of 13.5% was found, which considerably exceeds the current unemployment rate in Germany, which lay at 6.4% in 2015 [37]. It appears that – despite partly high formal qualifications and a share of 49.0% of participants having never experienced times of unemployment – adults with ASD often experience problems in maintaining stable employment relationships. In this regard, international studies suggest, that challenges in maintaining employment in adults with ASD can be explained by social and sensory issues interfering with their job performance as well as their ability to manage interactional aspects of work [4–6]. In view of the fact that 17.0% of those participants who had ever been employed later retired early for health reasons, and taking into account the reported high prevalence of comorbidities, it would appear that employment support is necessary that takes into account the specific characteristics of ASD regarding social and sensory issues, as well as further health-related aspects regarding the ability to work.

Although we found a strong trend towards occupations within the area “natural sciences, geography and computer sciences” compared to the general population [23], adults with ASD cover a broad range of occupational areas. In particular, the occupational area “health and social sector, teaching and education” was represented by a high proportion of our sample. This result does not tie in with the stereotype that adults with ASD are only found in more technically orientated, and not socially orientated, professions. These findings are in line with a study by Baldwin et al. [15] who also found that adults with ASD are employed in a wide variety of different occupational environments.

Even though the proportion of participants who were inadequately employed and overeducated was lower than that found in similar studies [12, 15], a significant difference emerged compared to the general population regarding the current occupation, with 31.3% of participants being inadequately employed in terms of overeducation. Moreover, the proportion of participants who were overeducated in their current profession was higher than the proportion of participants who were overeducated in their longest practised profession (22.1%). Thus, it appears that adults with ASD are partly unable to maintain professional positions in accordance with their level of formal qualification. This finding can be linked to studies, showing that differences with employers, i.e. regarding the understanding of productivity requirements and self-organization of work tasks, or possible ASD-related issues, i.e. impaired ability to handle social aspects, can hinder successful participation in the labour market [5–10].

According to our findings, while adults with ASD as seen in our specialised outpatient clinic have above-average levels of formal education and training, at least in part, they experience problems and challenges to establish themselves in the German labour market and/or to sustain professional positions that are appropriate to their level of formal education and training. This is alarming and in need of explanation. It can be assumed that the constraints associated with ASD are better compensated in educational contexts, as potentially impaired executive and soft-skills meet with higher tolerance, and individuals with ASD experience more assistance than is provided in working environments. Furthermore, greater flexibility and autonomy for individuals to utilize their own style of learning and working, with reduced impact of social difficulties, may also be a factor in the higher attainment of individuals with ASD in educational context compared with their employment status. This underlines the need for a better integration and improved support of adults with ASD in occupational settings, and demonstrates that potential strengths associated with ASD, such as honesty, efficiency or precision [38], should be identified and reinforced. This is of particular relevance since support programs for adults with ASD, addressing, e.g., work preparation and communication with employers, proved to be effective in increasing employment rates [39, 40]. A promising approach to meet the support requirements of adults with ASD could be “training-on-the-job” measures, as the most important issue does not seem to lie in obtaining professional positions, but rather in maintaining them. For the development and implementation of such measures, further research on the experience of employment situations in adults with ASD is needed, i.e. expectations regarding workplace design, challenges in professional transitions or experiences with ASD related sensory and interactional difficulties in occupational environments as well as support needs concerning these matters.

## Conclusions

Despite an above-average level of general education in terms of school-leaving qualifications and a high level of formal education and training, even exceeding the level within the general population, adults with late-life diagnosis of ASD are at a disadvantage regarding their participation in the labour market in Germany. Consequently, there are high rates of unemployment and early retirement for health reasons represented in our sample. Furthermore, adults with ASD seem to struggle to maintain professional positions that are appropriate to their formal qualifications and are often inadequately employed in terms of overeducation. Based on the results of our study, and on previous studies demonstrating good outcomes from employment support programs, the development of “training-on-the-job” interventions seems particularly promising to enhance the labour market participation of adults with ASD.

## Additional file

Additional file 1: Questionnaire. (PDF 145 kb)

## Abbreviations

ASD: Autism spectrum disorder
DSM-IV: Diagnostic and Statistical Manual of Mental Disorders 4th Revision
ICD-10: International Statistical Classification of Diseases and Related Health Problems 10th Revision
IQ: Intelligence quotient
KldB: Classification of occupation 2010
NICE: National Institute for Health and Care Excellence
