# Supplementary material for: Education and employment status of adults with autism spectrum disorders in Germany – a cross-sectional-survey
Source: BMC Psychiatry. 2018 Mar 27;18:75. doi: 10.1186/s12888-018-1645-7 (PMC5870494; doi:10.1186/s12888-018-1645-7)
Supplement: Supplementary file 1 — Questionnaire. (PDF 145 kb) [file 12888_2018_1645_MOESM1_ESM.pdf]

# Menschen mit

# Autismus-Spektrum-Störungen

# und der Arbeitsmarkt

**Bitte senden Sie den vollständig ausgefüllten Fragebogen mit beiliegendem frankiertem Rückumschlag an:**

Universitätsklinikum Freiburg
Klinik für Psychiatrie und Psychotherapie
Arbeitsgruppe Psychotherapie- und Versorgungsforschung
z. Hd. Herrn Dr. phil. Lars P. Hölzel
Hauptstraße 5, 79104 Freiburg

**Bei Fragen oder Rückmeldungen wenden Sie sich bitte an:**

Universitätsklinikum Freiburg
Klinik für Psychiatrie und Psychotherapie
Sektion Psychotherapieforschung in der Psychiatrie
Herrn Dr. phil. Thomas Fangmeier
Tel.: +49 (0)761/270-69680
E-Mail: thomas.fangmeier@uniklinik-freiburg.de

Liebe Teilnehmerin, lieber Teilnehmer,

im Folgenden finden Sie einige Hinweise und Erläuterungen zum Ausfüllen des Fragebogens. Bitte lesen Sie zuerst die Erklärungen zum Ausfüllen auf dieser Seite aufmerksam durch, bevor Sie mit dem Beantworten der Fragen beginnen. Die Arbeitszeit, die Sie zum Ausfüllen des Fragebogens benötigen, beträgt ca. 45-60 Minuten. Der Fragebogen kann auch in Etappen ausgefüllt werden.

Bitte füllen Sie den Fragebogen komplett aus.

Kreuzen Sie bei jeder Frage die Antwort an, die am ehesten auf Sie zutrifft.

Um eine Antwort zu markieren, setzen Sie bitte ein Kreuz in das Kästchen, welches am ehesten Ihrer Antwort entspricht, z. B.:

| überhaupt nicht | an einzelnen Tagen | an mehr als der Hälfte der Tage | beinahe jeden Tag |
|---|---|---|---|
| ☐ | ☐ | ☒ | ☐ |

Falls die aufgeführten Antworten Ihre Meinung nicht genau treffen, kreuzen Sie bitte die Antwort an, die am ehesten auf Sie zutrifft.

Wenn nicht anders vermerkt, ist **jeweils nur 1 Kästchen** als Antwort anzukreuzen. Andernfalls wird extra darauf hingewiesen, z.B. durch den Hinweis: **(Mehrfachantworten möglich)**. Fragen, bei denen mehrere Antworten angekreuzt werden können, erkennen Sie zudem daran, dass statt eines Kästchens ein Kreis zum Ankreuzen vor den möglichen Antworten steht.

Falls Sie versehentlich ein falsches Kreuz gemacht haben, streichen Sie das Kästchen bitte durch und kreuzen Sie die für Sie richtige Antwort an, z.B.:

| überhaupt nicht | an einzelnen Tagen | an mehr als der Hälfte der Tage | beinahe jeden Tag |
|---|---|---|---|
| ~~☐~~ | ☒ | ☐ | ☐ |

Wenn keine Fragen mehr bestehen, können Sie nun auf der nächsten Seite mit der Beantwortung des Fragebogens beginnen. Wenn Sie noch Fragen haben, wenden Sie sich bitte an Herrn Dr. phil. Thomas Fangmeier (Kontaktdaten auf dem Deckblatt).

## A: Schulischer und beruflicher Werdegang

Zunächst möchten wir Sie bitten, einige Fragen zu Ihrem **schulischen** und Ihrem **beruflichen Werdegang** zu beantworten.

A1. Welchen höchsten allgemeinbildenden **Schulabschluss** haben Sie?

*(Kreuzen Sie nur das Kästchen für die Antwort an, die am ehesten auf Sie zutrifft.)* (DEGS D-123)

- ☐ Haupt- oder Volksschulabschluss
- ☐ Realschulabschluss / Mittlere Reife / Fachschulreife
- ☐ POS (Polytechnische Oberschule) bzw. 10. Klasse (vor 1965: 8.Klasse)
- ☐ Fachhochschulreife / Abschluss einer Fachoberschule
- ☐ Abitur, allgemeine oder fachgebundene Hochschulreife
- ☐ Anderen Schulabschluss (z.B. im Ausland erworben)
- ☐ Schule beendet ohne Schulabschluss
- ☐ Noch keinen Schulabschluss

A2. Machen Sie zurzeit eine **Schulausbildung**?

*(Kreuzen Sie nur das Kästchen für die Antwort an, die am ehesten auf Sie zutrifft.)* (DEGS D-124)

- ☐ Nein ➢ ***Bitte weiter mit Frage A3***
- ☐ Ja, und ich strebe folgenden Schulabschluss an:
  - ☐ Hauptschulabschluss
  - ☐ Realschulabschluss / Mittlere Reife / Fachschulreife
  - ☐ Fachhochschulreife / Abschluss einer Fachoberschule
  - ☐ Abitur, allgemeine oder fachgebundene Hochschulreife
  - ☐ Anderen Schulabschluss

A3. Welchen höchsten beruflichen **Ausbildungsabschluss bzw. Hochschulabschluss** haben Sie?

*(Kreuzen Sie nur das Kästchen für die Antwort an, die am ehesten auf Sie zutrifft).* (DEGS D-125)

- ☐ Noch in beruflicher Ausbildung (Auszubildende/r, Studentin/Student, Berufsvorbereitungsjahr)
- ☐ Keinen Berufsschulabschluss und nicht in beruflicher Ausbildung
- ☐ Ausbildung an einer Fachschule, Meister-, Technikerschule, Berufs- oder Fachakademie abgeschlossen
- ☐ Beruflich-betriebliche Ausbildung (Lehre) abgeschlossen
- ☐ Beruflich-schulische Ausbildung abgeschlossen (Berufsfachschule, Handelsschule, Vorbereitungsdienst für den Mittleren Dienst in der öffentlichen Verwaltung)
- ☐ Anderen Ausbildungsabschluss
- ☐ Bachelor an (Fach-) Hochschule abgeschlossen
- ☐ Sonstigen Abschluss an einer Fachhochschule oder Ingenieurschule
- ☐ Sonstigen Abschluss an einer Universität oder Hochschule

A4. Machen Sie zurzeit eine **berufliche Ausbildung oder ein Studium**?

*(Kreuzen Sie nur das Kästchen für die Antwort an, die am ehesten auf Sie zutrifft.)* (angelehnt an DEGS D-126)

☐ Nein ➢ ***Bitte weiter mit Abschnitt B***

☐ Ja, es handelt sich um folgende Art der Ausbildung:

- ☐ Beruflich-betriebliche Ausbildung (Lehre)
- ☐ Beruflich-schulische Ausbildung (z.B. Ausbildung an Berufsfachschule, Handelsschule, Vorbereitungsdienst für den Mittleren Dienst in der öffentlichen Verwaltung)
- ☐ Besuch einer Fachschule, Meister-, Technikerschule, Berufs- oder Fachakademie
- ☐ Hochschul- oder Fachhochschulstudium
- ☐ Andere Ausbildung
- ☐ Berufsvorbereitende Maßnahmen

## B: Berufstätigkeit und Arbeitslosigkeit

Im folgenden Fragenblock werden nähere Angaben zu Zeiten der **Berufstätigkeit** und **Arbeitslosigkeit** erfragt.

B1. Waren Sie jemals **berufstätig**? Hierzu zählen sowohl Tätigkeiten im Angestelltenverhältnis als auch selbständige Tätigkeiten.

*(Kreuzen Sie nur das Kästchen für die Antwort an, die am ehesten auf Sie zutrifft.)*

☐ Nein ➢ ***Bitte weiter mit Abschnitt H***

☐ Ja

B2. Sind Sie zurzeit **berufstätig**?

*(Kreuzen Sie nur das Kästchen für die Antwort an, die am ehesten auf Sie zutrifft.)*

☐ Nein ➢ ***Bitte weiter mit Frage B5***

☐ Ja ➢ In welchem zeitlichen Umfang?

*(Mehrfachantworten möglich.)*

- ○ Vollzeit
- ○ Teilzeit, und zwar ____ %
- ○ Geringfügig (450-Euro- oder Minijob)
- ○ „Ein-Euro-Job“ (Bezug von ALG II)
- ○ Gelegentlich oder unregelmäßig beschäftigt
- ○ Berufliche Ausbildung / Lehre mit Ausbildungsvergütung
- ○ Vorübergehend freigestellt / beurlaubt ➢ ***Bitte weiter mit Frage B5***

| | |
|---|---|
| B3. | Sind Sie zur Zeit in einer Werkstatt für Menschen mit Behinderung (WfbM) tätig?<br>*(Kreuzen Sie nur das Kästchen für die Antwort an, die am ehesten auf Sie zutrifft.)* |

☐ Ja ☐ Nein

| | |
|---|---|
| B4. | Wie viele Stunden arbeiten Sie normalerweise <u>pro Woche</u>?<br>*(Bitte tragen Sie die Anzahl der Stunden auf dem dafür vorgesehenen Strich ein.)* (DEGS D-128) |

________ Arbeitsstunden pro Woche

| | |
|---|---|
| B5. | Trifft eine der folgenden Angaben auf Ihre <u>derzeitige berufliche Situation</u> zu?<br>*(Hier können mehrere Antworten angekreuzt werden.)* (angelehnt an DEGS D-131) |

- ○ Altersteilzeit (unabhängig davon, in welcher Phase befindlich)
- ○ Wehr- oder Bundesfreiwilligendienst, Freiwilliges Soziales / Ökologisches Jahr
- ○ In Rente / Pension
- ○ Hausfrau/-mann
- ○ Umschulung
- ○ Arbeitslos
- ○ Praktikum / Volontariat
- ○ Elternzeit
- ○ Nichts davon trifft zu

| | |
|---|---|
| B6. | Wie viele Monate waren Sie insgesamt in den <u>letzten 5 Jahren</u> **arbeitslos**?<br>*(Bitte tragen Sie die Anzahl der Monate auf dem dafür vorgesehenen Strich ein.)* (DEGS D-134) |

Insgesamt ________ Monate arbeitslos

| | |
|---|---|
| B7. | Sind Sie in **Rente** bzw. pensioniert?<br>*(Kreuzen Sie nur das Kästchen für die Antwort an, die am ehesten auf Sie zutrifft.)* (DEGS D-136) |

- ☐ Nein ➢ ***Bitte weiter mit Frage B9***
- ☐ Altershalber in Rente / pensioniert ➢ ***Bitte weiter mit Abschnitt H***
- ☐ Vorzeitig in Rente / pensioniert (**nicht aus gesundheitlichen Gründen**, sondern z.B. Modell der Altersteilzeit) ➢ ***Bitte weiter mit Frage B8***
- ☐ Aktuell **aus gesundheitlichen Gründen** in Rente / pensioniert ➢ ***Bitte weiter mit Frage B8***

| | |
|---|---|
| B8. | <u>Seit wann</u> sind Sie **vorzeitig berentet?**<br>*(Bitte geben Sie Monat und Jahr an der gekennzeichneten Stelle an.)* (DEGS D-137) |

__ __ / __ __ __ __

B9. Beziehen Sie eine gesetzliche…?

*(Kreuzen Sie nur das Kästchen an, das am ehesten für Sie passt.)* (angelehnt an DEGS D-138)

- ☐ Berufsunfähigkeitsrente (bis Dezember 2000)
- ☐ Erwerbsunfähigkeitsrente (bis Dezember 2000)
- ☐ Ich beziehe keine der genannten Renten.
- ☐ Rente wegen teilweiser Erwerbsminderung (ab Januar 2001)
- ☐ Rente wegen voller Erwerbsminderung (ab Januar 2001)

## C. Berufliche Tätigkeit

Die folgenden Fragen beziehen sich auf die **Art Ihrer beruflichen Tätigkeit**. Bitte antworten Sie stichwortartig und formulieren Sie so präzise wie möglich.

C1. Welche **berufliche Tätigkeit** haben Sie in Ihrem bisherigen Arbeitsleben am längsten ausgeübt?

Bitte benennen Sie Ihre **berufliche Tätigkeit**. Geben Sie bitte die **genaue Bezeichnung** beziehungsweise den **Fachbereich** an und benennen **auch den Wirtschaftszweig**, also z.B.:

- Berufliche Tätigkeit: Ärztin / Arzt; Fachbereich: Onkologie; Wirtschaftszweig: Gesundheitswesen
- Berufliche Tätigkeit: Fachinformatikerin / Fachinformatiker; Fachbereich: Anwendungsentwicklung; Wirtschaftszweig: Software-Branche

*(Bitte tragen Sie Ihre Antwort in den Kasten ein.)*

| Berufliche Tätigkeit: | Fachbereich: | Wirtschaftszweig: |
|---|---|---|
| | | |

C2. Welche **berufliche Tätigkeit** üben Sie derzeit hauptsächlich aus?

Bitte benennen Sie Ihre **berufliche Tätigkeit**. Geben Sie bitte die **genaue Bezeichnung** beziehungsweise den **Fachbereich** an und benennen **auch den Wirtschaftszweig**, also z.B.:

- Berufliche Tätigkeit: Ärztin / Arzt; Fachbereich: Onkologie; Wirtschaftszweig: Gesundheitswesen
- Berufliche Tätigkeit: Fachinformatikerin / Fachinformatiker; Fachbereich: Anwendungsentwicklung; Wirtschaftszweig: Software-Branche

*(Bitte tragen Sie Ihre Antwort in den Kasten ein.)*

| Berufliche Tätigkeit: | Fachbereich: | Wirtschaftszweig: |
|---|---|---|
| | | |

## D: Arbeitszufriedenheit

Die folgenden Fragen beziehen sich darauf, ob Sie mit Ihrem aktuellen oder Ihrem letzten Arbeitsplatz **zufrieden** sind bzw. waren.

☐ Bitte setzen Sie hier ein Kreuz, wenn Sie aktuell keinen Arbeitsplatz haben und sich Ihre nachfolgenden Angaben in Frage D1 und D2 auf Ihren letzten Arbeitsplatz beziehen.

D1. **Wie zufrieden** sind bzw. waren Sie mit dieser Tätigkeit?

*(Kreuzen Sie nur das Kästchen für die Antwort an, die am ehesten auf Sie zutrifft.)*

| Sehr zufrieden | Eher zufrieden | Eher unzufrieden | Überhaupt nicht zufrieden |
|---|---|---|---|
| ☐ | ☐ | ☐ | ☐ |

D2. Bitte wählen Sie aus den untenstehenden Arbeitsbedingungen beziehungsweise **Aspekten Ihres Arbeitsplatzes** diejenigen aus, **mit welchen Sie unzufrieden sind bzw. waren**.

*(Hier können mehrere Antworten angekreuzt werden.)*

- ○ Art der Tätigkeit
- ○ Kommunikationsschwierigkeiten mit der vorgesetzten Person oder Kolleginnen / Kollegen
- ○ Kein eigenes Büro
- ○ Zu wenig Unterstützung bei inhaltlichen Fragen
- ○ Zu wenig Rückmeldung in Bezug auf geleistete Arbeit
- ○ Zu wenig Unterstützung bei der Kommunikation mit anderen Menschen, z.B. Durchführung von Telefonaten nur dann, wenn unbedingt nötig
- ○ Zu viel äußere Reize, z.B. Geräusche oder helle, flackernde Lichtquellen
- ○ Zu viel direkter Kontakt zu Kolleginnen / Kollegen
- ○ Unklare Vorgaben über die zu leistende Arbeit
- ○ Zu langer Arbeitsweg
- ○ Keine ausreichende Einarbeitungsphase zu Beginn des Arbeitsverhältnisses
- ○ Zu geringes Gehalt
- ○ Zu niedrige eigene hierarchische Stellung
- ○ Zu geringe Wertschätzung und Respekt der eigenen Person
- ○ Zu viel direkter Kontakt zu externen Personen, z.B. Klientinnen- / Klienten-Kontakt, Käuferinnen- / Käufer- Kontakt oder Verkäuferinnen- / Verkäufer-Kontakt
- ○ Zu wenig Flexibilität bei den Arbeitszeiten
- ○ Sonstiges:

  *(Bitte tragen Sie Ihre Antwort in den Kasten ein.)*

## E: Schwierigkeiten am Arbeitsplatz

Es kann sein, dass es manchmal zu **Schwierigkeiten bei der Arbeit** kommt, die z.B. zu Konflikten im Team bis hin zu einem Verlust des Arbeitsplatzes führen können. Die Fragen in diesem Abschnitt beziehen sich auf Ihre Erfahrungen.

E1. Sind Sie in den letzten 12 Monaten, in denen Sie gearbeitet haben, durch eine/n Kollegin / Kollegen / Vorgesetzte/n am Arbeitsplatz **abgewertet worden** (z.B. bezüglich Ihres Aussehens, Ihrer Art sich zu kleiden, Ihrer Denk-, Handlungs- oder Arbeitsweise oder möglicher Behinderungen)? Oder wurden Sie beleidigt, beschimpft, bedroht, schikaniert oder unter Druck gesetzt?

*(Kreuzen Sie nur das Kästchen für die Antwort an, die am ehesten auf Sie zutrifft.)*

(angelehnt an DEGS D-24)

☐ Nein ➢ ***Bitte weiter mit Frage E2***

☐ Ja ➢ Wie stark fühlten oder fühlen Sie sich dadurch in Ihrem Befinden beeinträchtigt?

- ☐ Gar nicht
- ☐ Kaum
- ☐ Etwas
- ☐ Stark
- ☐ Sehr stark

E2. Haben Sie in der Vergangenheit schon mal einen **Arbeitsplatz verloren**?

*(Kreuzen Sie nur das Kästchen für die Antwort an, die am ehesten auf Sie zutrifft.)*

☐ Nein ➢ ***Bitte weiter mit E3***

☐ Ja ➢ Welche Gründe gab es dafür?

*(Bitte tragen Sie diese stichpunktartig in den untenstehenden Kasten ein.)*

E3. Welche **autismusspezifischen Verhaltensweisen** gibt es, die Ihrer Meinung nach bei Ihnen Ihre **Arbeit negativ beeinflussen** können?

*(Bitte tragen Sie diese stichpunktartig in den Kasten ein.)*

## F: Unterstützungsbedarf in der Bewerbungsphase und bei der Arbeit

In diesem Abschnitt wird danach gefragt, in welchen **Phasen des Prozesses der Arbeitssuche bis hin zur Situation am Arbeitsplatz** Sie **Unterstützung** benötigen. Dabei beziehen sich die Antworten nicht darauf, durch wen oder was Sie Unterstützung in Anspruch nehmen könnten, sondern darauf, **in welchem Bereich** Sie überhaupt **Unterstützungsbedarf** sehen.

Es wird zwischen vier zeitlichen Phasen unterschieden: Der erste Teil bezieht sich dabei auf die **Phase der Berufsorientierung**, der zweite Teil auf die **Phase der Bewerbung**. Der dritte Teil thematisiert den Unterstützungsbedarf bei der **Vorbereitung auf ein Bewerbungsgespräch** und der vierte Teil den Unterstützungsbedarf **am Arbeitsplatz**.

### F1. Unterstützungsbedarf in der Phase der Berufsorientierung

| *(Kreuzen Sie nur das Kästchen für die Antwort an, die am ehesten auf Sie zutrifft.)* | Keine Unter-stützung | Etwas Unter-stützung | Viel Unter-stützung | Sehr viel Unter-stützung |
|---|---|---|---|---|
| 1. Bei der Identifikation der eigenen Fähigkeiten | ☐ | ☐ | ☐ | ☐ |
| 2. Bei der Identifikation von passenden Berufsbildern | ☐ | ☐ | ☐ | ☐ |
| 3. Bei der Organisation von Hilfsangeboten für die Bewerbungsphase | ☐ | ☐ | ☐ | ☐ |

### F2. Unterstützungsbedarf in der Phase der Bewerbung

| *(Kreuzen Sie nur das Kästchen für die Antwort an, die am ehesten auf Sie zutrifft.)* | Keine Unter-stützung | Etwas Unter-stützung | Viel Unter-stützung | Sehr viel Unter-stützung |
|---|---|---|---|---|
| 1. Bei der Suche nach geeigneten Stellenanzeigen | ☐ | ☐ | ☐ | ☐ |
| 2. Beim Verfassen von Bewerbungen | ☐ | ☐ | ☐ | ☐ |
| 3. Beim Umgang mit der Diagnose Autismus-Spektrum-Störung im Rahmen der Bewerbung | ☐ | ☐ | ☐ | ☐ |

### F3. Unterstützungsbedarf bei der Vorbereitung auf ein Bewerbungsgespräch

| *(Kreuzen Sie nur das Kästchen für die Antwort an, die am ehesten auf Sie zutrifft.)* | Keine Unter-stützung | Etwas Unter-stützung | Viel Unter-stützung | Sehr viel Unter-stützung |
|---|---|---|---|---|
| 1. Bei der Zusammenstellung geeigneter Kleidung und z.B. der Auswahl einer angemessenen Frisur für das Bewerbungsgespräch | ☐ | ☐ | ☐ | ☐ |
| 2. Bei der inhaltlichen Vorbereitung auf ein Bewerbungsgespräch, z.B. der Überlegung, welche Fragen gestellt werden könnten | ☐ | ☐ | ☐ | ☐ |

| | *(Kreuzen Sie nur das Kästchen für die Antwort an, die am ehesten auf Sie zutrifft.)* | Keine Unter-stützung | Etwas Unter-stützung | Viel Unter-stützung | Sehr viel Unter-stützung |
|---|---|---|---|---|---|
| 3. | Bei dem Training von möglichen Gesprächssituationen innerhalb des Bewerbungsgesprächs Z. B.: Wie setze ich meine Stimme gewinnbringend ein? Wie funktioniert ein „normaler“ Blickkontakt? Wie beachte ich alle anwesenden Personen? Wie erkenne ich geltende soziale Regeln? | ☐ | ☐ | ☐ | ☐ |

| F4. | **Unterstützungsbedarf am Arbeitsplatz** | | | | |
|---|---|---|---|---|---|
| | *(Kreuzen Sie nur das Kästchen für die Antwort an, die am ehesten auf Sie zutrifft.)* | Keine Unter-stützung | Etwas Unter-stützung | Viel Unter-stützung | Sehr viel Unter-stützung |
| 1. | Beim Erkennen geltender sozialer Regeln | ☐ | ☐ | ☐ | ☐ |
| 2. | Bei der Einhaltung geltender sozialer Regeln | ☐ | ☐ | ☐ | ☐ |
| 3. | Bei der Einschätzung, wie intensiv eine Aufgabe erledigt werden sollte | ☐ | ☐ | ☐ | ☐ |
| 4. | Bei der Priorisierung von Aufgaben, z.B. durch Erstellung von Tages- und Wochenplänen | ☐ | ☐ | ☐ | ☐ |
| 5. | Beim Erkennen von frühen Anzeichen von abwertendem Verhalten durch Kolleginnen / Kollegen (Mobbing) | ☐ | ☐ | ☐ | ☐ |
| 6. | Bei der Gestaltung von Kontakten, z.B. Kundenkontakten | ☐ | ☐ | ☐ | ☐ |
| 7. | Bei der Organisation von externen Hilfsangeboten, falls Unsicherheiten bei der Arbeit auftreten, z.B. durch Job Coach, Selbsthilfegruppe, Autismus-Verein oder eine/n Autismustherapeutin / -therapeuten | ☐ | ☐ | ☐ | ☐ |
| 8. | Bei der Äußerung von Unklarheiten bei Aufgabenstellungen | ☐ | ☐ | ☐ | ☐ |

| *(Kreuzen Sie nur das Kästchen für die Antwort an, die am ehesten auf Sie zutrifft.)* | | Keine Unter-stützung | Etwas Unter-stützung | Viel Unter-stützung | Sehr viel Unter-stützung |
|---|---|---|---|---|---|
| 9. | Bei Konflikten innerhalb des Teams | ☐ | ☐ | ☐ | ☐ |
| 10. | Beim Umgang mit Gefühlen der Überforderung aufgrund von zu erledigenden Aufgaben | ☐ | ☐ | ☐ | ☐ |
| 11. | Beim Umgang mit Gefühlen der Überforderung aufgrund von sozialen Anforderungen | ☐ | ☐ | ☐ | ☐ |
| 12. | Beim Umgang mit Misserfolgen durch nicht korrekt gelöste Aufgaben | ☐ | ☐ | ☐ | ☐ |
| 13. | Bei der Veranlassung der Einrichtung eines reizarmen Arbeitsplatzes | ☐ | ☐ | ☐ | ☐ |
| 14. | Beim Umgang mit Veränderungen am Arbeitsplatz | ☐ | ☐ | ☐ | ☐ |
| 15. | Beim Verfassen von E-Mails, z.B. durch einen Leitfaden | ☐ | ☐ | ☐ | ☐ |
| 16. | Bei der Durchführung von Telefongesprächen, z.B. durch einen Leitfaden oder ein Training von möglichen Gesprächssituationen | ☐ | ☐ | ☐ | ☐ |
| 17. | Bei der Einschätzung eigener Kompetenzen und Entscheidungsspielräume | ☐ | ☐ | ☐ | ☐ |
| 18. | Sonstiges: Hier ist Platz, um weitere, für Sie wichtige Formen der Unterstützung rund um den Arbeitsplatz einzugeben.<br><br>*(Bitte tragen Sie diese stichpunktartig in den Kasten auf der rechten Seite ein.)* | | | | |

## G: Schwierigkeiten aufgrund von Gesundheitsproblemen

Im Folgenden geht es um Schwierigkeiten, die aufgrund von Gesundheitsproblemen entstehen können. Gesundheitsprobleme beinhalten Krankheiten oder Erkrankungen, andere Gesundheitsprobleme, die lang oder kurz andauernd sein können, Verletzungen, mentale oder emotionale Probleme sowie Drogen- oder Alkoholprobleme.

Denken Sie bitte an die letzten 30 Tage zurück und beantworten Sie bitte die folgenden Fragen im Hinblick darauf, **wie viele Schwierigkeiten** Sie bei der Durchführung der nachfolgenden Aktivitäten hatten. Kreuzen Sie bei jeder Frage nur eine Antwort an.

*(Kreuzen Sie bei jeder Frage bitte nur eine Antwort an. Lassen Sie bitte keine Aussage aus und kreuzen Sie im Zweifelsfall die Antwortmöglichkeit an, die noch am ehesten auf Sie zutrifft.)*

(WHODAS-s)

| | *Wie viele Schwierigkeiten hatten Sie in den letzten 30 Tagen:* | Keine | Geringe | Mäßige | Starke | Sehr starke/ nicht möglich |
|---|---|---|---|---|---|---|
| 1. | Eine längere Zeit (ca. 30 Minuten) zu stehen? (S1) | ☐ | ☐ | ☐ | ☐ | ☐ |
| 2. | Ihren Haushaltspflichten nachzukommen? (S2) | ☐ | ☐ | ☐ | ☐ | ☐ |
| 3. | Neue Aufgaben zu lernen (z.B. Erlernen an einen neuen Ort zu gelangen, den Sie nicht kannten)? (S3) | ☐ | ☐ | ☐ | ☐ | ☐ |
| 4. | Wie viele Schwierigkeiten hatten Sie, an gesellschaftlichen Aktivitäten (wie z.B. Festlichkeiten, religiöse oder andere Aktivitäten) in der gleichen Art und Weise teilzunehmen, wie jeder andere? (S4) | ☐ | ☐ | ☐ | ☐ | ☐ |
| 5. | Wie sehr wurden Sie durch Ihren gesundheitlichen Zustand emotional belastet? (S5) | ☐ | ☐ | ☐ | ☐ | ☐ |
| 6. | Sich für 10 Minuten auf etwas zu konzentrieren? (S6) | ☐ | ☐ | ☐ | ☐ | ☐ |
| 7. | Eine längere Strecke (ca. einen Kilometer) zu Fuß zu gehen? (S7) | ☐ | ☐ | ☐ | ☐ | ☐ |
| 8. | Ihren gesamten Körper zu waschen? (S8) | ☐ | ☐ | ☐ | ☐ | ☐ |

| | *Wie viele Schwierigkeiten hatten Sie in den letzten 30 Tagen:* | Keine | Geringe | Mäßige | Starke | Sehr starke/ nicht möglich |
|---|---|---|---|---|---|---|
| 9. | Sich anzuziehen? (S9) | ☐ | ☐ | ☐ | ☐ | ☐ |
| 10. | Im Umgang mit Personen, die Sie nicht kennen? (S10) | ☐ | ☐ | ☐ | ☐ | ☐ |
| 11. | Eine Freundschaft aufrechtzuerhalten? (S11) | ☐ | ☐ | ☐ | ☐ | ☐ |
| 12. | Bei der Bewältigung Ihres Arbeits- /Schulalltags? (S12) | ☐ | ☐ | ☐ | ☐ | ☐ |

Bei den nächsten drei Antworten wird kein Kästchen angekreuzt, sondern auf dem Strich die **Anzahl der Tage** angegeben.

| | | |
|---|---|---|
| 13. | An wie vielen Tagen traten diese Schwierigkeiten während der letzten 30 Tage auf? (H1) | Anzahl der Tage: ______________________________ |
| 14. | An wie vielen Tagen in den letzten 30 Tagen waren Sie aufgrund Ihrer Gesundheitsprobleme absolut unfähig, alltägliche Aktivitäten oder Ihre Arbeit zu verrichten? (H2) | Anzahl der Tage: ______________________________ |
| 15. | An wie vielen Tagen in den letzten 30 Tagen mussten Sie aufgrund Ihrer Gesundheitsprobleme alltägliche Aktivitäten oder Ihre Arbeit reduzieren? (H3) | Anzahl der Tage: ______________________________ |

## H: Verschiedene Aspekte von Sprache

Im Folgenden finden Sie einige Aussagen über **verschiedene Aspekte von Sprache**. Bitte lesen Sie sich die Aussagen durch und entscheiden Sie, inwieweit die jeweilige Aussage auf Sie zutrifft.

In manchen Sätzen wird auch der Begriff **„Metapher“** benutzt. Eine Metapher ordnet einer Sache einen anderen Namen als üblich zu, um gedanklich ein bestimmtes Bild zu erzeugen, welches Ähnlichkeit mit der Sache hat (z.B. werden die Äste und Blätter eines Baumes wegen ihrer äußeren Form auch als Baumkrone bezeichnet).

*(Kreuzen Sie bei jeder Frage bitte nur eine Antwort an. Lassen Sie bitte keine Aussage aus und kreuzen Sie im Zweifelsfall die Antwortmöglichkeit an, die noch am ehesten auf Sie zutrifft.)*

(FQLP)

| | | Ich stimme zu. | Ich stimme eher zu. | Ich stimme eher nicht zu. | Ich stimme nicht zu. |
|---|---|---|---|---|---|
| 1. | Mein Sprachverständnis unterscheidet sich von dem anderer Menschen. | ☐ | ☐ | ☐ | ☐ |
| 2. | Ich verstehe oft nicht, was andere mir sagen wollen. | ☐ | ☐ | ☐ | ☐ |
| 3. | Im Gespräch finde ich Metaphern und / oder Sprichwörter irritierend. | ☐ | ☐ | ☐ | ☐ |
| 4. | Mir nicht bekannte Metaphern und / oder Sprichwörter erschließen sich mir intuitiv. | ☐ | ☐ | ☐ | ☐ |
| 5. | Metaphern und / oder Sprichwörter halte ich für unnötig. | ☐ | ☐ | ☐ | ☐ |
| 6. | Ich erkenne nicht-wörtlich gemeinte Ausdrücke daran, dass ich sie in der Vergangenheit schon einmal gehört und damals missverstanden habe. | ☐ | ☐ | ☐ | ☐ |
| 7. | Ironie erkenne ich meistens ohne Probleme. | ☐ | ☐ | ☐ | ☐ |
| 8. | In der Schulzeit habe ich Aussagen meiner Lehrer und Mitschüler häufig missverstanden. | ☐ | ☐ | ☐ | ☐ |
| 9. | Ich habe bewusst daran gearbeitet, Metaphern / Sprichwörter besser zu verstehen. | ☐ | ☐ | ☐ | ☐ |
| 10. | Ich erschließe mir die Bedeutungen von Metaphern etc. durch rationales Analysieren. | ☐ | ☐ | ☐ | ☐ |
| 11. | In einer idealen Sprache gibt es keine Zwei- oder Mehrdeutigkeiten. | ☐ | ☐ | ☐ | ☐ |

## I: Angaben zur Person

Abschließend möchten wir Sie bitten, einige **Informationen zu Ihrer Person** anzugeben.
Ihre persönlichen Daten werden nur in Gruppen ausgewertet dargestellt, so dass keine Rückschlüsse auf Ihre Person möglich sind.

**I1.** Wie alt sind Sie?

*(Bitte runden Sie Ihr Alter auf volle Jahre auf oder ab.)*

__________ Jahre

**I2.** Welches Geschlecht haben Sie?

(DEGS D-2)

- ☐ Weiblich
- ☐ Männlich

**I3.** Welchen Familienstand haben Sie?

*Mit „verheiratet“ sind auch eingetragene Lebenspartnerschaften gemeint.* (DEGS D-119)

- ☐ Verheiratet, mit Ehepartnerin / Ehepartner zusammen lebend
- ☐ Geschieden
- ☐ Verheiratet, von Ehepartnerin / vom Ehepartner getrennt lebend
- ☐ Verwitwet
- ☐ Ledig

**I4.** Haben Sie eine/n feste/n Partnerin / Partner, mit der / dem Sie mindestens sechs Monate zusammen sind?

(DEGS D120)

- ☐ Ja
- ☐ Nein

**I5.** Haben Sie eine oder mehrere der folgenden lang andauernden, chronischen Krankheiten? Chronische Krankheiten sind lang andauernde Erkrankungen, die ständiger Behandlung und Kontrolle bedürfen, z.B. Diabetes, Herzerkrankungen oder Zwangserkrankungen.

*(Hier können mehrere Antworten angekreuzt werden.)* (angelehnt an DEGS D-4)

- ☐ Nein ➢ ***Bitte weiter mit Frage I6***
- ☐ Ja, und zwar:
  - ○ Depressive Störung
  - ○ Zwangserkrankung
  - ○ Orthopädische Problematik (z.B. chronischer Rückenschmerz)
  - ○ Herzerkrankung
  - ○ Atemwegserkrankungen
  - ○ Diabetes
  - ○ Sonstiges: *(Bitte nutzen Sie für Ihre Antwort den Kasten.)*

I6. Welche Form der Autismus-Spektrum-Störung ist bei Ihnen diagnostiziert worden?

*(Kreuzen Sie nur das Kästchen für die Antwort an, die am ehesten auf Sie zutrifft.)*

- ☐ Frühkindlicher Autismus (F84.0)
- ☐ Atypischer Autismus (F84.1)
- ☐ Asperger Autismus (F84.5)
- ☐ Tief greifende Entwicklungsstörung, nicht näher bezeichnet (F84.9)
- ☐ Ich kenne meine Diagnose nicht.

I7. Mit wie vielen Jahren ist bei Ihnen die Autismus-Spektrum-Störung erstmalig durch einen Arzt diagnostiziert worden?

Mit ________ Jahren

J1. Wurde der Fragebogen selbständig ausgefüllt?

- ☐ Ja
- ☐ Nein. Mir hat geholfen:

*(Bitte tragen Sie Ihre Antwort in den Kasten ein und geben die Funktion der helfenden Person an, z.B. „Familienangehörige/r“.)*

**K: Anmerkungen und Vorschläge**

Haben Sie **Anmerkungen oder Vorschläge** zu unserer Studie oder diesem Fragebogen? Hier ist Platz dafür.

## Vielen Dank für Ihre Mitarbeit und die Unterstützung unserer Studie!
